# Endothelial toll‐like receptor 4 maintains lung integrity via epigenetic suppression of p16^INK4a^

**DOI:** 10.1111/acel.12914

**Published:** 2019-02-20

**Authors:** So‐Jin Kim, Peiying Shan, Cheol Hwangbo, Yi Zhang, Jin‐Na Min, Xuchen Zhang, Taylor Ardito, Alfred Li, Tien Peng, Maor Sauler, Patty J. Lee

**Affiliations:** ^1^ Pulmonary, Critical Care and Sleep Medicine, Department of Internal Medicine Yale University School of Medicine New Haven Connecticut; ^2^ Division of Applied Life Science (BK21 Plus), PMBBRC, Division of Life Science, College of National Sciences Gyeongsang National University Jinju Korea; ^3^ Department of Pathology Yale University School of Medicine New Haven Connecticut; ^4^ Bone Imaging Research Core University of California, San Francisco (UCSF) San Francisco California; ^5^ Department of Medicine, Cardiovascular Research Institute UCSF San Francisco California

**Keywords:** aging, cellular senescence, emphysema, HDAC2, p16^INK4a^, toll‐like receptor 4

## Abstract

We previously reported that the canonical innate immune receptor toll‐like receptor 4 (TLR4) is critical in maintaining lung integrity. However, the molecular mechanisms via which TLR4 mediates its effect remained unclear. In the present study, we identified distinct contributions of lung endothelial cells (Ec) and epithelial cells TLR4 to pulmonary homeostasis using genetic‐specific, lung‐ and cell‐targeted in vivo methods. Emphysema was significantly prevented via the reconstituting of human TLR4 expression in the lung Ec of TLR4−/− mice. Lung Ec‐silencing of TLR4 in wild‐type mice induced emphysema, highlighting the specific and distinct role of Ec‐expressed TLR4 in maintaining lung integrity. We also identified a previously unrecognized role of TLR4 in preventing expression of p16^INK4a^, a senescence‐associated gene. Lung Ec‐p16^INK4a^‐silencing prevented TLR4−/− induced emphysema, revealing a new functional role for p16^INK4a^in lungs. TLR4 suppressed endogenous p16^INK4a^ expression via HDAC2‐mediated deacetylation of histone H4. These findings suggest a novel role for TLR4 in maintaining of lung homeostasis via epigenetic regulation of senescence‐related gene expression.

## 1 INTRODUCTION

Emphysema, identified histologically as lung airspace enlargement, occurs in the lung as a consequence of advanced age and/or chronic obstructive pulmonary disease (COPD). COPD is a major leading cause of morbidity and mortality worldwide (Mannino & Buist, 2007). Advancing age is one of the most common risk factors, regardless of smoking status, for the development of COPD (Fukuchi, 2009). Nonsmoke exposed, aged wild‐type (WT) mice, including the widely used C57Bl6 mice, exhibit emphysema, suggesting a biologic relationship between aging and emphysema (Huang, Rabold, Schofield, Mitzner, & Tankersley, 2007).

Cellular senescence is a state of irreversible growth arrest provoked by multiple mechanisms such as telomere shortening, DNA damage, or oncogene activation (Campisi & di Fagagna, 2007). Cell cycle arrest, a feature of cell senescence, is considered to be an adaptive mechanism to prevent damaged cells from undergoing aberrant proliferation (Campisi & di Fagagna, 2007). This state of proliferative arrest is due to p16^INK4a^/retinoblastoma (pRb) and/or p14^ARF^/p53 pathways (Vandenberk, Brouwers, Hatse, & Wildiers, 2011). p16^INK4a^ inhibits complex of cyclin‐dependent kinase 4‐6 (CDK4‐6)/cyclin D, thereby preventing phosphorylation of Rb protein family, leading to G1 phase cell cycle arrest (Vandenberk et al., 2011). Several hallmarks of cellular senescence include upregulation of CDK inhibitors, such as p16^INK4a^_,_ p21^Cip1/Waf1^, and acquisition of senescence‐associated β‐galactosidase activity (SA‐β‐gal; Campisi, 2013). In addition, the increased production of SA secretory phenotype (SASP), such as interleukin (IL)‐1α, IL‐6, IL‐8, granulocyte–macrophage colony‐stimulating factor (GM‐CSF), monocyte chemoattractant protein (MCP) 1, 2, and matrix metalloproteinase, is also a well‐known characteristic of cellular senescence (Campisi, 2013). Senescence is closely associated with the loss of tissue and organ function in aging tissues (Campisi, 2013). A high level of p16^INK4a^ is reported in the alveolar epithelium of people with COPD (Muñoz‐Espín & Serrano, 2014). Consistently, increased expression of p16^INK4a^ and p21^Cip1/Waf1^ in endothelial cells (Ec) and alveolar type was opposite to that of the proliferation marker PCNA (Tsuji, Aoshiba, & Nagai, 2006). However, the regulatory function of p16^INK4a^ in lungs remains poorly understood.

Epigenetic changes, such as histone acetylation/deacetylation, have been closely linked with cellular senescence and have been detected in human COPD (Sundar, Nevid, Friedman, & Rahman, 2014). Histone deacetylases (HDAC) regulate gene expression by catalyzing the removal of acetyl groups from the lysine tails of core histones. Interestingly, decreased HDAC expression correlated with an increase in histone acetylation was reported in senescent cells (Wagner et al., 2001). HDAC2 belongs to the class I family and plays a critical role in suppressing inflammatory gene expression in the airways, lung parenchyma, and airspace macrophages (Barnes, 2009). In addition, HDAC2 controls G1 to S transition and its absence provokes a senescence‐like G1 cell cycle arrest (Noh et al., 2011). Also, reduced HDAC2 expression and activity have been associated with human COPD (Ito et al., 2005).

We previously revealed new roles for the innate immune receptor, toll‐like receptor (TLR)4, in maintaining lung integrity at baseline as well as during acute oxidant injury (Takyar, Haslip, Zhang, Shan, & Lee, 2016; Zhang, Shan, Jiang, Cohn, & Lee, 2006; Zhang et al., 2005), specifically in lung Ec. Our current studies offer novel mechanistic insights by linking TLR4‐p16^INK4a^‐mediated cellular senescence. TLR4 is a member of a diverse family of pattern‐recognition receptors, which activate the innate immune response and subsequently induce anti‐microbial adaptive immunity (Medzhitov, 2001). In human emphysema, reduced TLR4 expression has been associated with more severe emphysema and airflow obstruction (Lee et al., 2012; Speletas et al., 2009). Although these human studies confirmed the clinical relevance of TLR4 deficiency in human COPD, the molecular mechanisms remain unclear.

Here, we sought to delineate nonmicrobial roles for TLR4 in preventing emphysema using tissue‐ and cell‐specific targeting of TLR4 in vivo, and to translate our findings to primary human lung microvascular Ec (hLMVEC). To the best of our knowledge, our studies are the first to demonstrate that Ec TLR4 suppresses cellular senescence through HDAC2‐histone H4‐p16^INK4a^. We demonstrate that lungs and Ec deficient in *TLR4* gene exhibit a senescence phenotype in vivo and that lung‐targeted silencing of Ec‐p16^INK4a^ prevents age‐related emphysema in TLR4−/− mice. These studies reveal mechanistic links between innate immunity, senescence, and emphysema while identifying potentially new therapeutic targets for age‐related lung diseases.

## 2 RESULTS

### 2.1 TLR4 deficiency in mice leads to age‐related emphysema

**Figure 1:**
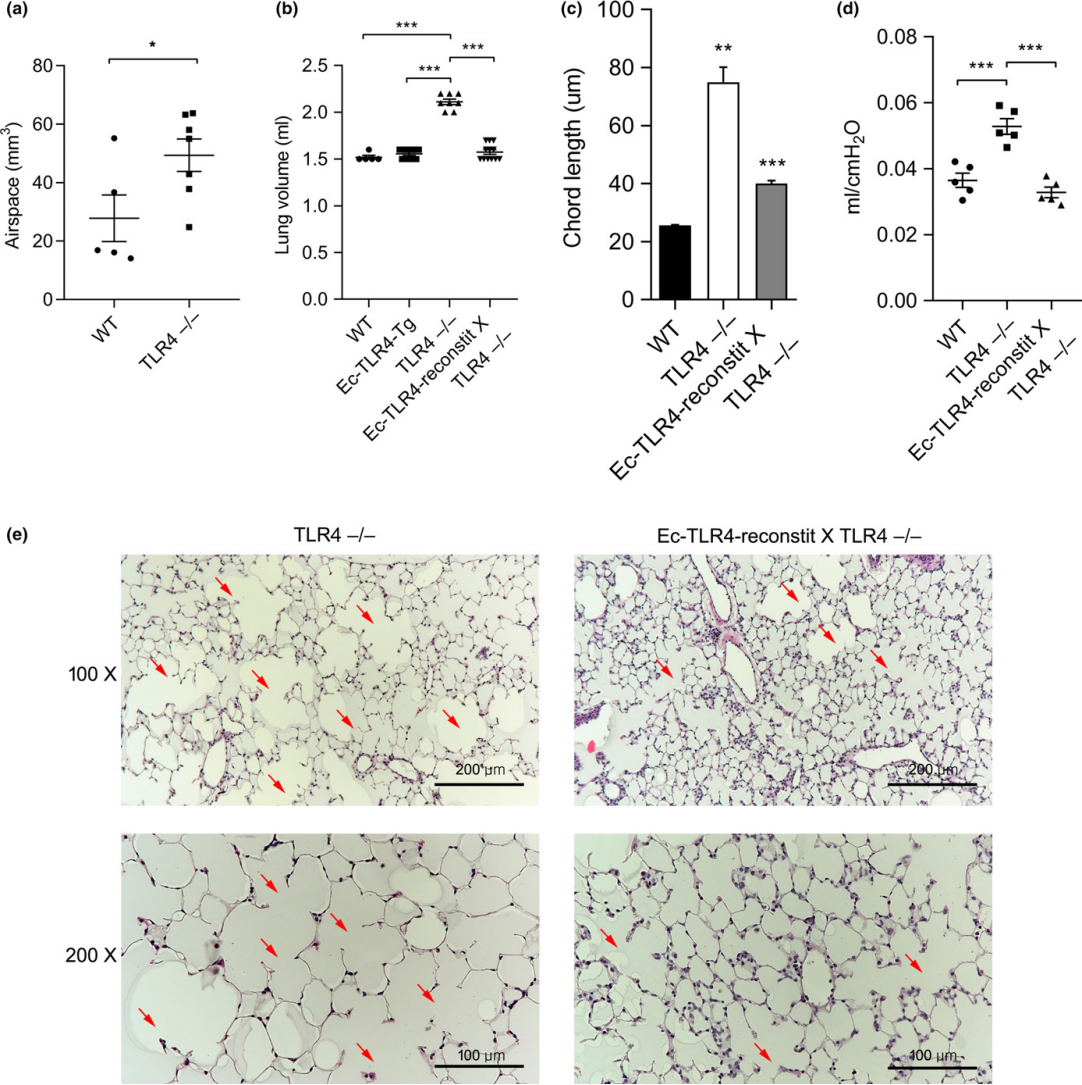
Endothelial (Ec) TLR4 prevents age‐related emphysemain in TLR4 ‒/‒ mice. (a) µCT analysis of murine lungs in wild‐type (WT) and TLR4−/− mice. (n = 5 and 7, respectively) *p < 0.05. (b) Lung volumes at 3 months of age from WT, Ec‐TLR4‐transgenic (Tg), TLR4−/−, and Ec‐TLR4‐reconstitution (reconstit) X TLR4−/− mice. (n = 5, 9, 8, and 12, respectively) ***p < 0.001. (c) Mean chord lengths (n = 3 per group) and (d) lung compliance (n = 5 per group) at 3 months of age from WT, TLR4−/−, and Ec‐TLR4‐reconstit X TLR4−/− mice. **p < 0.01, ***p < 0.001. (e) Lung histology of TLR4−/− and Ec‐TLR4‐reconstit X TLR4−/− mice. Arrows point to airspace enlargement

We previously reported that a physiologic consequence of TLR4 deficiency is postdevelopmental, age‐related spontaneous emphysema, and identified increased oxidant production via NADPH oxidase 3, as a potential mechanism in lung Ec (Zhang et al., 2006, 2016). To further understand the role of TLR4 in age‐related emphysema, we extended the time course to older ages up to 12 months and found that TLR4−/− mice exhibited increased lung volumes and chord lengths at significantly earlier ages than age‐matched WT mice (Supporting Information Figure S1A,B). Of note, mild, age‐related lung enlargement in WT mice occurs only after 18–20 months (data not shown). Lung compliance measurements on FlexiVent, which is considered to be a specific measurement of emphysema, were also increased in TLR4−/− (Supporting Information Figure S1C; Vanoirbeek et al., 2010). These data are confirmed by histologic evidence of airspace enlargement in TLR4−/− mice (Supporting Information Figure S1D). We also performed µCT of murine lungs of both WT and TLR4−/−, followed by ex vivo 3D lung volume reconstruction techniques, as we recently reported (Wang et al., 2018), to quantify the percentage of airspace volume. TLR4−/− mice showed significantly increased airspace volume compared to WT, (Figure 1a).

### 2.2 **Reconstituting Ec‐TLR4 in TLR4**−/−** mice prevents age‐related emphysema**

Based on our previous report that TLR4 bone marrow reconstitution had no impact on emphysema (Zhang et al., 2006) or survival during oxidant lung injury (Takyar et al., 2016), we focused on TLR4 reconstitution in structural cells rather than circulating cells. We selected Ec as our first target based on our previous reports that lung Ec responses were critical to lung maintenance and resistance to injury. We proceeded to generate mice that express the human TLR4 (hTLR4) transgene, in the presence of DOX, only in Ec (Ec‐TLR4‐reconstit) to confirm the role of Ec TLR4 in modulating age‐related emphysema in TLR4 ‐/‐ mice. We confirmed that reconstituted mice expressed hTLR4 by qPCR and in situ hybridization with *hTLR4* targeting primers (Supporting Information Figure S2A,B). Consistent with previous data, TLR4−/− mice showed enlarged lung volumes at 3 months old, which was completely prevented in Ec‐TLR4‐reconstit X TLR4−/− mice (Figure 1b). Consistent with these data, Ec‐TLR4‐reconstition mice prevented increases in chord length and compliance in TLR4−/−, indicating that Ec is a key compartment in preventing emphysema (Figure 1c,d). These data were supported by histological observation of the lung tissues (Figure 1e). Epi‐TLR4‐reconstition also prevented emphysema but not to the full extent observed in Ec‐TLR4‐reconstit (Supporting Information Figure S3). This suggests that there is likely an airway Epi‐TLR4 contribution but given the scope of the current studies, we chose to focus on Ec‐TLR4.

### 2.3 Silencing Ec‐TLR4 in WT mice is sufficient to cause emphysema in WT mice

**Figure 2:**
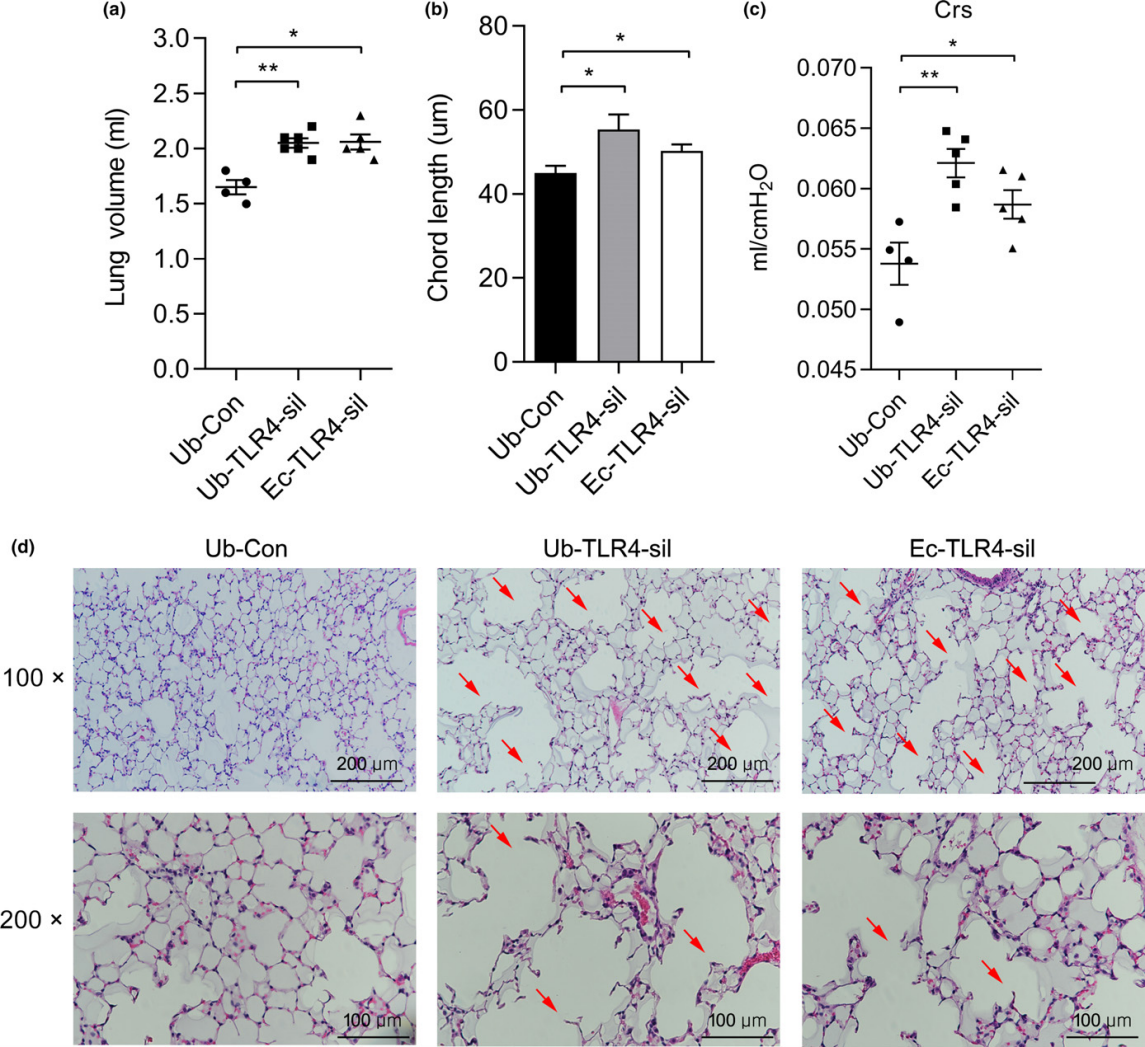
Endothelial cell (Ec)‐targeted TLR4 silencing (Ec‐TLR4‐sil) in WT lungs is sufficient to cause emphysema. (a) Lung volumes, (b) mean chord lengths, (c) lung compliance (Crs), and (d) H&E stained lung sections of WT mice after 2 months of initial intranasal administration of either Ubiquitin (Ub) or VE‐Cadherin (Ec)‐conjugated RNAi lentivirus with a control (Con) or TLR4 target sequence (Ub‐Con: n = 4, Ub‐TLR4‐sil: n = 6, Ec‐TLR4‐sil: n = 5) *p < 0.05, **p < 0.01 vs. Ub‐Con. Arrows point to enlarged airspaces

To determine the impact of TLR4 silencing specifically in lungs and in lung Ec, we constructed ubiquitin (Ub)‐ and Ec‐targeted lentiviral (Lenti) TLR4 silencing (Ub‐ and Ec‐TLR4‐sil, respectively) constructs and delivered them intranasally (to achieve lung‐targeting), per our previously reported lung‐targeted Lenti methods (Haslip et al., 2015; Takyar et al., 2016; Zhang et al., 2016). Ec‐TLR4‐sil was established by using the vascular endothelial cadherin construct, which is considered to have greater specificity for mature Ec. We confirmed 78% and 65% knockdown of *TLR4* gene in the lung Ec isolated from lung tissues of Ub‐ and Ec‐TLR‐sil mice, respectively (Supporting Information Figure S4). Intranasal delivery of control lentivirus (Ub‐Con) had no impact on lung volumes, while Ub‐TLR4‐sil resulted in lung enlargement after 3 months, like that of TLR4−/− mice (Figure 2a, middle groups). Ec‐TLR4‐sil appeared equally effective as Ub‐TLR‐sil, again suggesting the central role of Ec (Figure 2a, right groups). The lung chord lengths and compliance were also increased in both Ub‐ and Ec‐TLR4‐sil mouse (Figure 2b,c). Additionally, histologic evaluation revealed enlargement of the airspaces accompanied by loss of the normal airspace architecture in Ec‐TLR4‐sil mice and Ub‐TLR4‐sil, characteristic of emphysema (Figure 2d).

### 2.4 TLR4 deficiency increases cellular senescence

**Figure 3:**
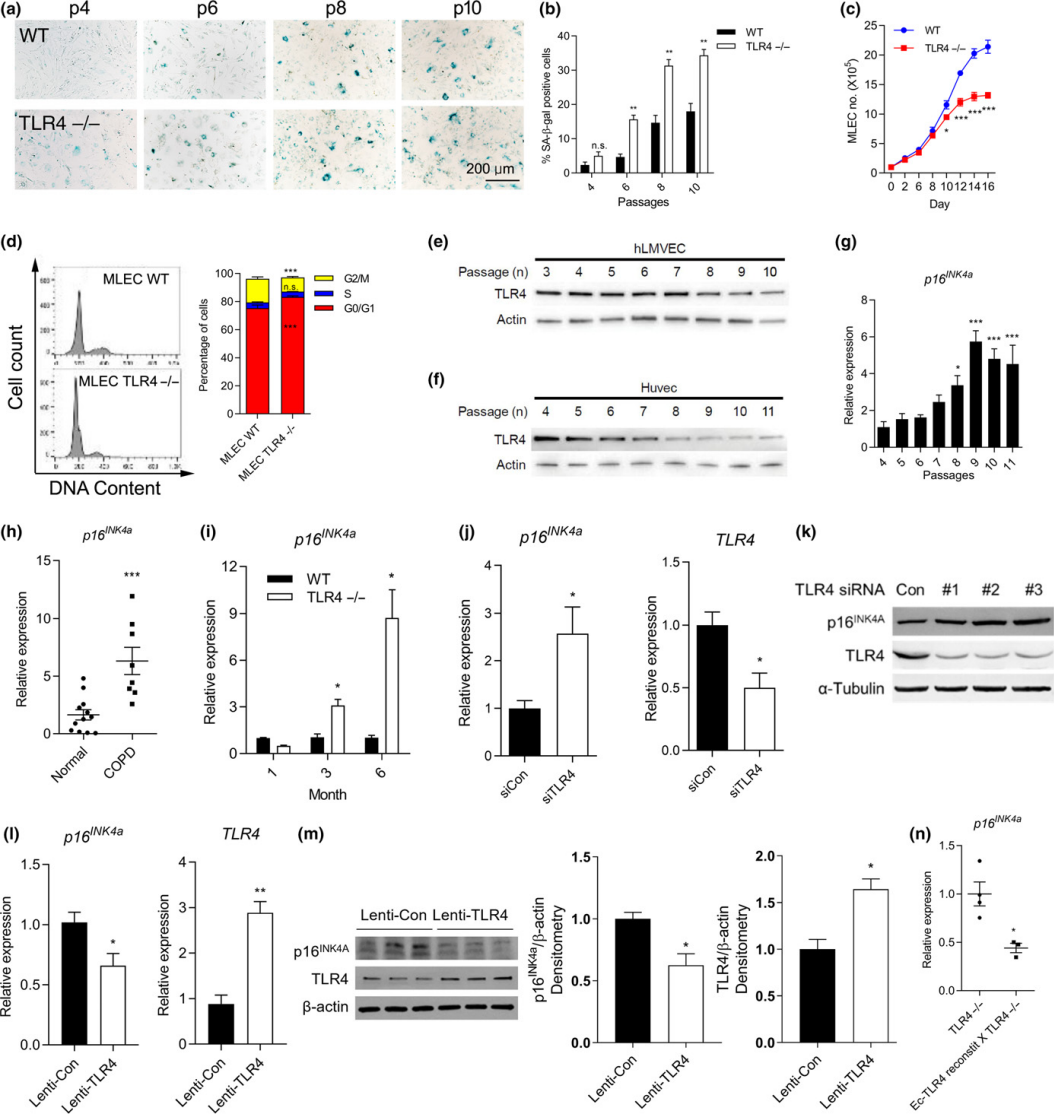
TLR4−/− induces cellular senescence in murine and human endothelial cells (Ec). (a) Representative images and (b) quantification of the senescence‐associated (SA)‐β‐gal activities in WT and TLR4−/− MLEC as passage numbers increase. Blue staining indicates SA‐β‐gal positive cells. **p < 0.01 vs. WT, n.s.: nonsignificant. (c) WT and TLR4−/− MLEC growth were observed over 16 days. (*n* = 3 in each group at different time points). *p < 0.05, ***p < 0.001 vs. WT. (d) FACS analysis of cell cycle for PI‐stained WT and TLR4−/− MLEC (*n* = 3 and *n* = 4, respectively). ***p < 0.001 vs. MLEC WT, n.s.: nonsignificant. TLR4 expression in (e) hLMVEC and (f) HUVEC at indicated passage numbers. (g) Expression of *p16^INK4a^* mRNA in HUVEC at indicated passage numbers. *p < 0.05, ***<0.001 vs. passage 4. (h) Expression of *p16^INK4a^* mRNA in lungs from normal and COPD subjects (*n* = 12 and 8, respectively). The mean ± *SEM* of ages are 59.5 ± 4.3 and 56.0 ± 1.8 in normal and COPD subjects, respectively. ***p < 0.001 vs. Normal. (i) *p16^INK4a^* mRNA expression in lungs at 1, 3, and 6 month of age from WT and TLR4−/− mice. (*n* = 3 per group) *p < 0.05 vs. age‐matched WT. (j) The mRNA and (k) protein expression of p16^INK4a^ and TLR4 in control (siCon)‐ or TLR4‐siRNA (siTLR4)‐treated hLMVEC. *p < 0.05 vs. siCon. (l) The mRNA and (m) protein expression of p16^INK4a^ and TLR4 in Lentiviral vector‐mediated hTLR4 overexpressed hLMVEC. *p < 0.05, **p < 0.01 vs. Lenti‐Con. (n) *p16^INK4a^* mRNA expression in lung homogenates of TLR4−/− and Ec‐TLR4‐reconstit X TLR4−/− mice. (WT: *n* = 4, *TLR4*−/−: *n* = 3). *p < 0.05 vs. TLR4−/−.

Human emphysema and COPD have been associated with induction of cellular senescence (Chilosi, Carloni, Rossi, & Poletti, 2013; Tsuji et al., 2006), and therefore, we asked the question of whether TLR4 deficiency‐mediated emphysema was also associated with cellular senescence. Consistent with the early‐onset of emphysema in TLR4−/− mice, we detected increased SA‐β‐gal activity, a marker for cellular senescence, in TLR4−/− mice lung compared to that in the WT lung at 6 month (Supporting Information Figure S5A,B). Also, TLR4−/− mouse lung Ec (MLEC) showed increase in SA‐β‐gal activity, even at low passages, compared to WT MLEC (Figure 3a,b). To further investigate the role of TLR4 in cellular senescence, we examined the cell growth rate and cell cycle analysis between TLR4−/− and WT MLEC. TLR4−/− MLEC had a significantly decreased rate of cell growth compared with WT MLEC (Figure 3c). Analysis of cell cycle distribution by flow cytometry showed that TLR4−/− MLEC exhibited increased G1 phase (83.4 ± 1.4% vs. 74.5 ± 3.8%) and decreased G2 phase (10.0 ± 1.5% vs. 16.8 ± 2.4%) compared to WT MLEC (Figure 3d). Using two types of primary human Ec, hLMVEC and HUVEC, we found TLR4 protein expression decreased after advanced passages (Figure 3e,f), while *p16^INK4a^* mRNA level increased in HUVEC (Figure 3g), suggesting that cellular aging correlates with decreased TLR4 expression. However, there were no differences in mRNA levels of *GM‐CSF*, *MCP1* and *2* between the groups (Supporting Information Figure S5C). We also found that Ec‐TLR4‐reconstit X TLR4−/− mice lungs showed significantly decreased *IL‐1α* and *IL‐6* mRNA expressions compared to TLR4−/− mice lungs (Supporting Information Figure S6). These results demonstrate that TLR4−/− mice exhibit early, progressive airspace enlargement as they age, and TLR4 deficiency is associated with markers of cell senescence, such as increased SA‐β‐gal and p16^INK4a^ expression, and decreased cell proliferation.

### 2.5 **TLR4 deficiency induces p16^INK4a^‐mediated** **cellular senescence**

Increased expression of p16^INK4a^ is a characteristic feature of senescence in human emphysema (Müller et al., 2006), but studies remain associative and its precise role in emphysema pathogenesis remains unclear. Total lung lysates from control and COPD human lungs (obtained from the lung tissue research consortium, LTRC) showed higher *p16^INK4a^* mRNA expression than controls (Figure 3h). We also found that TLR4−/− mice lungs showed significantly increased *p16^INK4a^* mRNA expression compared to WT mice at 3 and 6 months age (Figure 3i). We also measured mRNA levels of *p19^Arf^*, *p21^Cip1/Waf1^*, and *p53*. *p21^Cip1/Waf1^* was also increased in TLR4−/− mice lung (Supporting Information Figure S7A) but only *p16^INK4a^* decreased in Ec‐TLR4‐reconstit mice lung compared to TLR4−/− mice lung, which led us to focus on p16^INK4a^ *.* The expressions of *p19^Arf^*, *p21^Cip1/Waf1^,*and *p53*were unchanged (Supporting Information Figure S7B). WT lungs did not exhibit increased p16^INK4a^ in lungs until 18–24 month (data not shown).

Using a live‐cell detection method for *p16^INK4a^* mRNA expression, the PrimeFlow RNA assay, we confirmed our qPCR results. The average *p16^INK4a^* mRNA expression in live cells significantly increased in TLR4−/− MLEC compared to WT MLEC (Supporting Information Figure S8). Moreover, selective inhibition of TLR4 by siRNA in hLMVEC led to a significant increase in both p16^INK4a^ mRNA and protein expression (Figure 3j,k). In contrast, TLR4 overexpression resulted in a significant decrease in both p16^INK4a^ mRNA and protein expression in hLMVEC (Figure 3l,m). Interestingly, we found *p16^INK4a^* mRNA expression was suppressed in lungs from Ec‐TLR4‐reconstit X TLR4−/− mice compared to TLR4−/− (Figure 3n), suggesting that Ec‐TLR4 negatively regulates p16^INK4a^ expression in lungs that exhibit early emphysema. Furthermore, micro array analysis confirmed increased *p16^INK4a^* gene‐related pathways in TLR4−/− MLEC compared to that in the WT MLEC (Supporting Information Figure S9). The clinical relevance of TLR4 is supported in our observations that human lung Ec express decreased TLR4 protein at higher passages, an in vitro model of aging, and in our analyses of human COPD lungs.

### 2.6 **Lung‐Ec targeting of p16^INK4a^ prevents emphysema in TLR4**−/−** mice**

**Figure 4:**
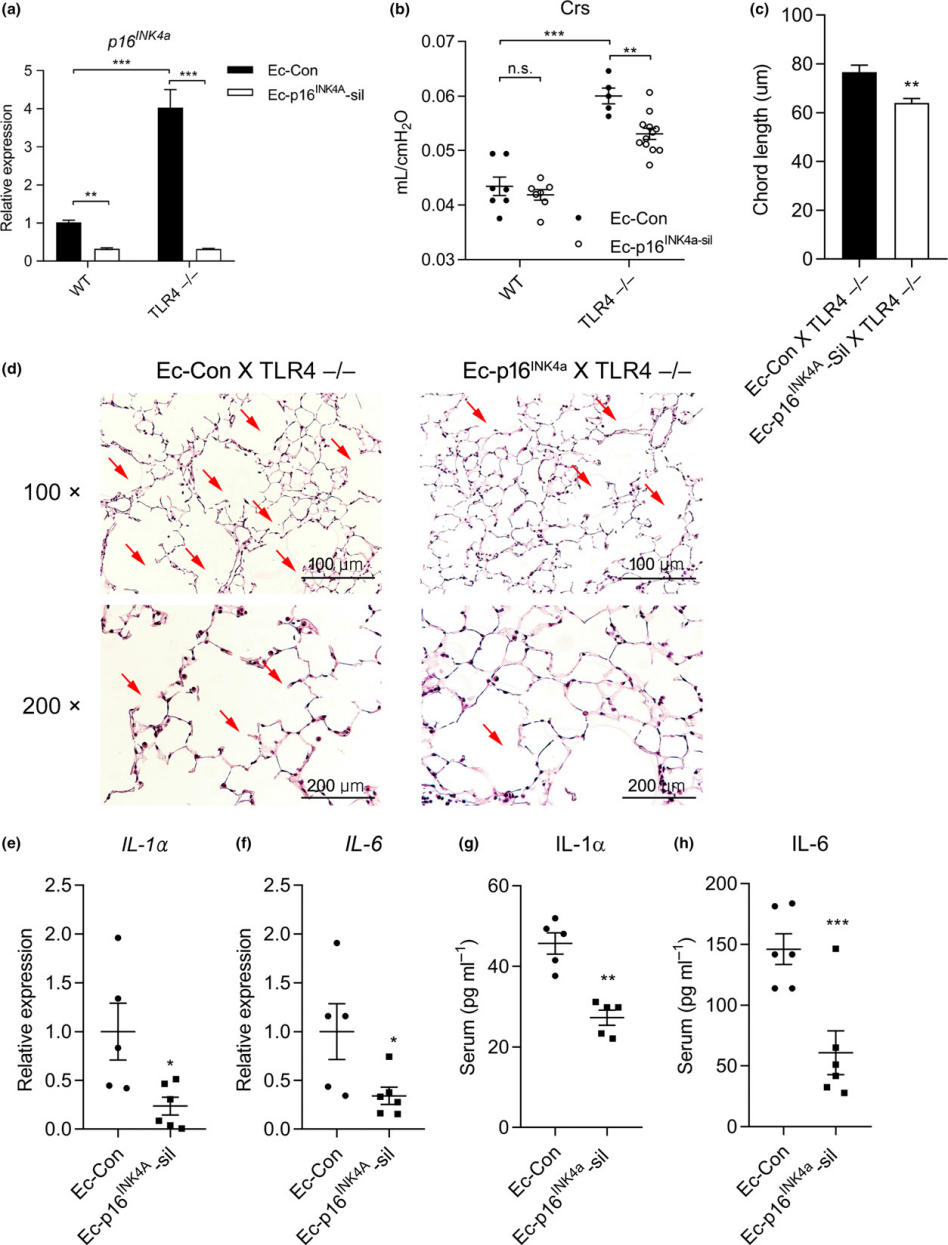
Lung endothelial cell (Ec)‐targeted p16^INK4a^ silencing (Ec‐p16^INK4a^‐sil) prevents emphysema in TLR4−/− mice. (a) p16^*INK4a*^ mRNA expression in Ec‐Con or Ec‐p16^INK4a^‐sil X WT or TLR4−/− mice. **p < 0.01, ***p < 0.001. (b) Lung compliance at 3 months of age after lung Ec‐targeted silencing of p16^INK4a^ in TLR4−/−. **p < 0.01, ***p < 0.001, n.s.: nonsignificant. (c) Mean chord lengths from Ec‐Con or Ec‐p16^INK4a^‐sil X TLR4−/−. (*n* = 3 per group) **p < 0.01 vs. Ec‐Con X TLR4−/−. (d) H&E stained lung sections taken from Ec‐Con or Ec‐p16^INK4a^‐sil X TLR4−/−. Arrows point to enlarged airspaces. (e) *IL‐1α* and (f) *IL‐6*mRNA expressions in Ec‐Con or Ec‐p16^INK4a^‐sil X TLR4−/− mice lung. (Ec‐Con XTLR4−/− mice: *n* = 5, Ec‐p16^INK4a^‐sil X TLR4−/− mice: *n* = 6) *p < 0.05 vs. Ec‐Con X TLR4−/−. (g) Serum IL‐1α (*n* = 5) and (h) IL‐6 (*n* = 6) concentration in Ec‐Con or Ec‐p16^INK4a^‐sil X TLR4−/− mice lung **p < 0.01, ***p < 0.001 vs. Ec‐Con X TLR4−/−

We sought to provide in vivo evidence of a functional role for p16^INK4a^ induction in lungs, which to our knowledge has not been previously reported. We selected a lung‐targeted Lenti silencing construct, using our Ec‐cell targeting VE‐Cad promoter (Ec‐p16^INK4a^‐sil), to determine whether Ec‐p16^INK4a^had any functional role in our model. We harvested lung‐derived cells from WT and TLR4−/− mice and then obtained single cell suspension with a magnetic‐activated cell sorting system. Among the cell population, Ec expressed the highest level of *p16^INK4a^*mRNA expression (Supporting Information Figure S10). We confirmed in vivo silencing efficiency by decreased *p16^INK4a^* mRNA expression, determined qPCR (Figure 4a). *p16^INK4a^*mRNA expression was suppressed in Ec‐p16^INK4a^‐sil X TLR4−/− mice lung compared to TLR4−/− mice (Supporting Information Figure S11A), but *p21^Cip1/Waf1^* mRNA expression was unchanged (Supporting Information Figure S11B). As expected, p16^INK4a^‐sil had no effect in WT lungs, while lung‐targeted Ec‐p16^INK4a^‐sil had a profound effect on preventing emphysema in 3 months old TLR4−/− mice, as measured by lung compliance (Figure 4b). These findings were confirmed by chord length (Figure 4c) and histologic evaluation (Figure 4d). Moreover, SASP components, *IL‐1α* and *IL‐6* mRNA expression (Figure 4e,f), and secreted IL‐1α and IL‐6 in serum (Figure 4g,h) were suppressed in Ec‐p16^INK4a^‐sil X TLR4−/− mice lungs compared to Ec‐Con X TLR4−/− mice lung. These data strongly suggest that not only is Ec‐p16^INK4a^ regulated by TLR4 in vivo, but lung Ec‐p16^INK4a^ regulates specific lung SASPs and is critical to the pathogenesis of age‐related emphysema in the setting of TLR4 deficiency.

### 2.7 **HDAC2 expression is decreased in TLR4**−/−** MLEC**

**Figure 5:**
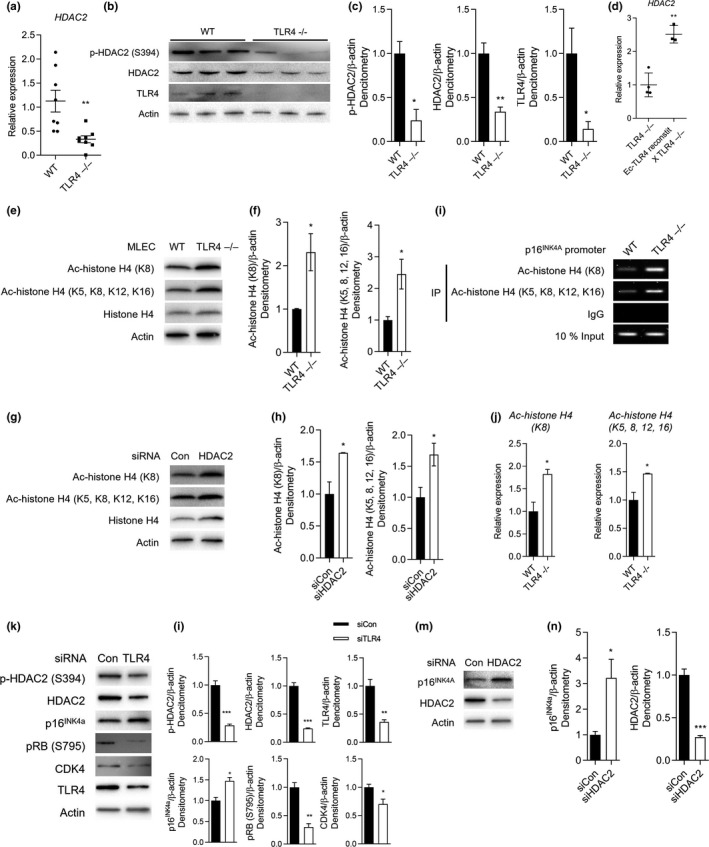
Endothelial cell (Ec)‐TLR4 suppresses p16^INK4a^ via histone deacetylase (HDAC)2 in mouse lung Ec (MLEC) and in lungs. (a) *HDAC2* mRNA expression in WT and TLR4−/− MLEC. (*n* = 7 per group) **p < 0.01 vs. WT. (b) Phosphorylated (p)‐HDAC2 and HDAC2 protein expressions in lung homogenates of WT and TLR4−/− mice, and (c) their representative graphs. (*n* = 3 per group). *p < 0.05, **p < 0.01 vs. WT. (d) *HDAC2* mRNA expression in lung homogenates of TLR4−/− and Ec‐TLR4‐reconstit X TLR4−/− mice. (e) Acetylated (Ac)‐histone H4 (K8 or K5, 8, 12, 16) and histone H4 protein expression in WT and TLR4−/− MLEC, and (f) their representative graphs (*n* = 3 per group). *p < 0.05 vs. WT. (g) Ac‐histone H4 (K8 or K5, 8, 12, 16) and histone H4 protein expression in HDAC2 siRNA (siHDAC2) in hLMVEC and (h) their representative graphs (*n* = 3 per group). *p < 0.05 vs. siCon. (i) Chromatin‐IP analysis for measuring relationship between p16^INK4a^ and Ac‐histone H4. IgG was used as a negative control, whereas 10% input DNA was used as a positive control. (j) Graph represents Ac‐histone H4 (K8)/10% input and Ac‐histone H4 (K5, 8, 12, 16)/10% input intensity ratio (*n* = 3 per group). *p < 0.05 vs. WT. (k) p‐HDAC2, HDAC2, p16^INK4a^, pRB (S795), CDK4 and TLR4 protein expression in siCon or siTLR4‐treated hLMVEC and (l) their representative graphs (*n* = 3 per group). *p < 0.05, **p < 0.01, ***p < 0.001 vs. siCon. (m) p16^INK4a^ and HDAC2 protein expression in siCon or siHDAC2‐treated hLMVEC and (n) their representative graphs (*n* = 3 per group). *p < 0.05, ***p < 0.001 vs. siCon

Previous reports have demonstrated that HDAC2 expression and activity are significantly reduced in human COPD (Qu, Yang, Ma, He, & Xiao, 2013), as well as in cigarette smoke (CS)‐exposed mouse lungs (Adenuga, Yao, March, Seagrave, & Rahman, 2009). As further evidence that TLR4‐deficiency recapitulates, to some extent, human COPD and CS‐induced emphysema, we found that *HDAC2* mRNA expression significantly decreased in lungs from TLR4−/− mice compared to WT (Figure 5a). We also found that phosphorylated (p)‐ and total‐HDAC2 protein expression were significantly decreased in TLR4−/− MLEC compared to WT (Figure 5b,c). We checked other HDAC isoforms, including Class I HDAC1 and HDAC3, Class II HDAC4 and HDAC6, and Class IV HDAC11, but these were not significantly changed in TLR4−/− mice (Supporting Information Figure S12). Importantly, *HDAC2* mRNA expression was restored in lungs of Ec‐TLR4‐reconstit X TLR4−/− mice, which supports a role for Ec‐TLR4 in regulating HDAC2 in vivo (Figure 5d).

### 2.8 TLR4 regulates HDAC2 expression and histone H4 acetylation

Others have reported that H4K8Ac in vivo significantly increased upon HDAC2 deletion (Hagelkruys et al., 2014). Although damage‐dependent H4K8Ac induction could occur through multiple mechanisms, we reasoned that it might reflect the action of HDAC2. Consequently, we tested whether the level of H4K8Ac is affected by TLR4. We found that H4K8Ac and H4K5, 8, 12, 16Ac were increased in TLR4−/− MLEC compared to WT MLEC (Figure 5e,f). Selective inhibition of HDAC2 by siRNA in hLMVEC led to significant increases in H4K8Ac and H4K5, 8, 12, 16Ac expressions (Figure 5g,h), demonstrating a TLR4‐HDAC2 axis in histone targets. We further performed chromatin immunoprecipitation (ChIP) assay to evaluate modifications specific to the p16^INK4a^ promoter. Higher levels of Ac‐histone H4 were bound to p16^INK4a^ promoter in TLR4 ‐/‐ MLEC compared to WT (Figure 5i,j). To confirm the regulatory role of TLR4 in HDAC2 and histone H4 protein/acetylation, we restored hTLR4 expression in TLR4−/− MLEC (Supporting Information Figure S13A). Lentiviral vector‐mediated hTLR4 (Lenti‐hTLR4) overexpression attenuated SASP induction, such as IL‐6 production in TLR4−/− MLEC (Supporting Information Figure S13B). In addition, Lenti‐hTLR4 overexpression decreased acetylation of histone H4 and increased HDAC2 protein expression (Supporting Information Figure S13C), supporting our hypothesis that Ec‐TLR4 regulates HDAC2 via histone acetylation.

### 2.9 TLR4 and HDAC2 regulate p16^INK4a^ expression

We then evaluated p16^INK4a^ expression after two different siRNA treatments. TLR4 siRNA significantly decreased p‐HDAC2 and HDAC2 protein expression and increased senescence, indicated by increased p16^INK4a^ and decreased pRB and CDK4 protein expression in hLMVEC (Figure 5k,l); as further confirmation, HDAC2 siRNA significantly increased p16^INK4a^ (Figure 5m,n). These data show that both TLR4 and HDAC2 modulate p16^INK4a^ expression, that the direction of modulation parallels what has been found in human COPD, and that HDAC2 is regulated by TLR4, suggesting that TLR4 prevents senescence, in part, by modulating HDAC2 activation.

## 3 DISCUSSION

TLRs mediate host defense by distinguishing pathogens from self. There is accumulating evidence that TLRs are activated by more than just pathogens and that their roles extend beyond pathogen recognition (Doz et al., 2008). We previously reported that mammalian TLR4 is required for survival during lethal oxidant stress injury or bleomycin‐induced lung injury (Jiang et al., 2005; Zhang et al., 2005), and that TLR4 deficiency causes a pro‐oxidant environment in tissues, as measured increased NADPH oxidase expression (Zhang et al., 2006). We also reported that TLR4 plays a crucial role in maintaining lung integrity, but the molecular mechanisms are unclear. In the present study, we confirmed that TLR4−/− mice were susceptible to age‐related emphysema. To determine which cell type is the greatest contributor of this protective effect, we developed cell‐specific TLR4 reconstitution in vivo. Ec‐TLR4‐reconstit completely prevented TLR4−/− induced increases in lung volume, chord lengths, and compliance, while Epi‐TLR4‐reconstit showed only partial effects. This result suggests the cell specificity of TLR4‐mediated pathways. Next, we investigated the role of lung‐specific Ec‐TLR4 silencing in emphysema. Because of lack of specific lung‐Ec promoters, we achieved lung Ec‐TLR4‐sil using intranasal lenti‐constructs delivery method, which we pioneered (Haslip et al., 2015; Takyar et al., 2016; Zhang, Jiang, Sauler, & Lee, 2013; Zhang et al., 2016). Of note, lung Ec‐TLR4‐sil mice showed increases in lung compliance and chord lengths, to a similar degree to Ubi‐TLR4‐sil mice, indicating that lung Ec‐TLR4‐sil is sufficient to cause emphysema. Our current findings support a previously unrecognized role of lung Ec‐TLR4 in maintaining normal lung integrity and thus preventing early emphysema.

Emphysema shares many of the cellular and molecular features associated with aging. Although CS is a common identifiable risk factor for emphysema/COPD, most smokers do not develop emphysema whereas advanced age is universally true in all nongenetic, nonfamilial cases of COPD. Thus, understanding the molecular links between aging and emphysema could lead to new therapeutic approaches. Cellular senescence, an irreversible cell cycle process, acts to protect against cancer, and more recently, this process is emerging as a regulator of complex biological processes such as development, tissue repair, and aging (van Deursen, 2014). For instance, ablation of p16^INK4a^ reverses the aging phenotype in α‐klotho, an aging suppressor gene, knockout mice (Sato et al., 2015), and brain Ec are reported as a susceptible cell type in BubR1 hypomorphic‐induced premature senescence mice, confirmed by increased SA‐β‐gal activity and p16^INK4a^ expression (Yamazaki et al., 2016). Recently, Baker et al. (2016) showed that p16^INK4a^ positive cells promote age‐dependent changes in organs such as kidney, heart, and fat, but notably these studies excluded lungs. Of note, CS‐induced emphysema/COPD in people has been associated with several markers of cellular senescence, such as p16^INK4a^(Aoshiba, Zhou, Tsuji, & Nagai, 2012). Despite these intriguing links, a pathophysiologic mechanism for cellular senescence in emphysema/COPD has not been firmly established. In the present study, people with COPD showed increased *p16^INK4a^* gene expression compared to that in the healthy controls. In addition, human Ec expressed more p16^INK4a^ expression with increasing passage numbers, and this increased p16^INK4a^ expression in Ec was correlated with decreased TLR4 expression. These results are confirmed by increased p16^INK4a^ expression in siTLR4‐treated Ec and decreased expression in TLR4‐overexpressed Ec. Hall et al. (2017) also reported that the stimulation of peritoneal macrophages with the TLR4 ligand, lipopolysaccharide significantly decreased SA‐β‐gal activity, indicating a protective effect of TLR4 against senescence. Collectively, TLR4 protects against age‐related emphysema, in part, via attenuating p16^INK4a^ expression.

Epigenetic changes are regarded as pathological mechanisms in age‐related disorders, including lung cancer and COPD (Vucic et al., 2014). Histone proteins act to pack or unpack DNA, which wraps around the eight histones, into chromosomes, also called histone posttranslational modification. Histone acetylation is a mode of epigenetic regulation mediated by histone acetyltransferases and HDACs, which catalyze the addition or removal of acetyl groups from acetyl‐CoA onto specific lysine residues, respectively. The lungs of people with COPD display increased rates of acetylated‐lysine (Su et al., 2013) as well as reduced HDAC2 expression and activities response to oxidative stress (Aoshiba et al., 2012). Of note, senescent cells showed decreased HDAC activity, corresponding with an increase in histone acetylation, and sodium dibutyrate, a HDAC inhibitor, induced p16^INK4a^ expression in human fibroblast cells (Munro, Barr, Ireland, Morrison, & Parkinson, 2004). We, therefore, hypothesized that HDAC may regulate TLR4‐mediated cellular senescence by histone acetylation modification. Among the five HDAC isotypes, only HDAC2 expression was decreased in TLR4−/− lung cells. Decreased HDAC2 expression has been reported to be associated with human COPD lungs (Qu et al., 2013), a pattern that we also detected in TLR4−/− Ec. Zheng, Li, Zhang, Balluff, and Pan (2012) reported that downregulation of p16^INK4a^ transcription in high fat diet‐fed mice was associated with reduced Ac‐histone H4, but this was not associated with Ac‐histone H3. On the other hand, Zhou, Han, Li, and Tong (2009) demonstrated the relationship between deacetylation of histone H3 and reduced p16^INK4a^ expression. In the present study, we found increased Ac‐histone H4 expression as well as its interaction with the p16^INK4a^ promoter in the absence of TLR4 in lung Ec. Collectively, these results suggest that TLR4 reduces p16^INK4a^ expression via HDAC2‐histone H4‐related epigenetic regulation.

The clinical relevance of this study is supported by data from our laboratory and others; (a) TLR4 expression is decreased in human COPD lungs (Lee et al., 2012; Speletas et al., 2009), (b) TLR4 expression decreases in Ec as they age, the most common risk factor for emphysema/COPD, and (c) many of the senescence and epigenetic molecular changes found in TLR4−/− lungs and Ec are also found in human COPD lungs (Ito et al., 2005; Qu et al., 2013; Tsuji et al., 2006). Lastly, we show a functional role for Ec‐p16^INK4a^ in emphysema by demonstrating that lung‐targeted Ec‐p16^INK4a^ in vivo blocks specific SASP induction and, importantly, prevents emphysema in TLR4−/− mice. Thus, p16^INK4a^ is not merely a marker, but a part of the pathogenetic mechanism of age‐related emphysema. Taken together, our studies reveal new molecular interactions between TLR4 and senescence and their functional impact on lung integrity.

**Figure 6:**
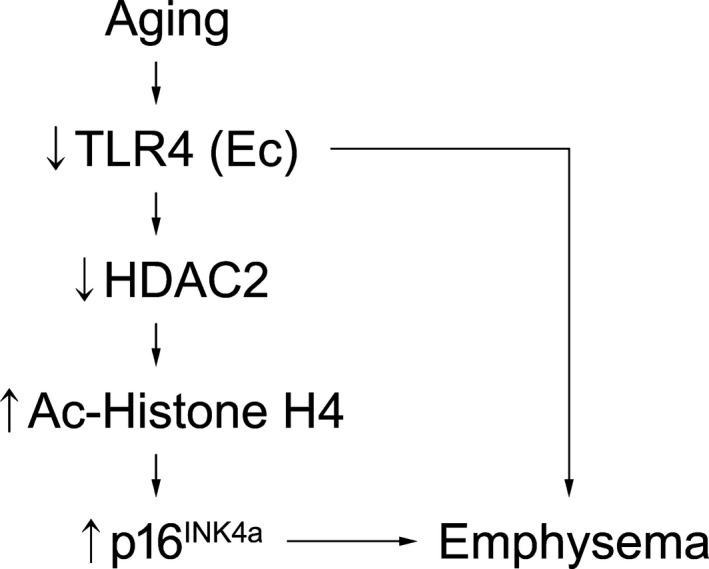
Summary scheme of proposed mechanism. Proposed mechanisms for TLR4‐mediated protection against age‐related emphysema via epigenetic suppression of p16^IN4a^ expression

In conclusion, our findings establish a novel signaling paradigm that, at least in part, reveal molecular relationships amongst innate immunity, aging, and lung maintenance while highlighting new functional roles for lung Ec (Figure 6). We also identify HDAC2 and p16^INK4a^ as potential therapeutic targets for emphysema. Future studies will fully explore the role of CS in accelerating senescence pathways and dissect the precise interactions amongst TLR4, HDAC2, and p16^INK4a^, which will offer additional molecular targets in treating a range of age‐related lung pathologies.

## 4 EXPERIMENTAL PROCEDURES

### 4.1 Human samples

Human lung DNA was obtained from the Lung Tissue Research Consortium (LTRC).

### 4.2 Mice

The research protocols were approved by the Institutional Animal Care and Use Committee of Yale University. C57Bl6 mice (Jackson Laboratories; Bar Harbor, ME) were maintained in the Animal Research Center at Yale University. We have previously described the TLR4−/−, Tie2‐TLR4‐Tg, CC10‐TLR4‐Tg, airway Epi‐Tg mice (Takyar et al., 2016). For Ec‐ or Epi‐TLR4‐reconstit X TLR4−/− mice, Tie2‐ or CC10‐TLR4‐Tg mice were bred with TLR4−/− mice, respectively. The DOX‐inducible mice were treated with 1 mg/ml DOX (DOT Scientific; Burton, MI) with 4% sucrose in drinking water (lightly shaded) starting at 4 weeks of age. For silencing of TLR4 expression in Ub or Ec in lung, WT mice were exposed to Ub‐ or Ec‐TLR4‐sil by intranasal administration. For silencing of p16^INK4a^ expression in lung Ec, WT mice were exposed to Ec‐p16^INK4a^‐sil by intranasal administration.

### 4.3 Pulmonary function test

A SCIREQ FlexiVent (SCIREQ; Montreal, Canada) ventilator was used to perform pulmonary function analyses on mice. Mice were tracheotomized with an endotracheal cannula while under anesthesia with 0.1 mg/kg of Urethan (U2500; Sigma; St. Louis, MO) intraperitoneally (i.p.), and connected to the FlexiVent ventilator. After mice were paralyzed with pancuronium bromide (0.1 mg/kg; i.p., Sigma, P1918), the lungs were ventilated with a Constant Flow ventilator, using the flexiWare software, which displays flow‐volume loops and automatically calculates volumes at sequentially increasing pressures as well as flow parameters. For each parameter, three measurements were measured and averaged. Serum was collected immediately after lung compliance measurements. All data were analyzed using flexiWare version 7.5, Service Pack 4 software (SCIREQ).

### 4.4 Chord lengths

After euthanasia, a cannula was inserted into the mouse trachea and secured with strings. The lungs were inflated with 1% agarose solution, which was first warmed to 45°C, and then immersed in ice‐cold 10% formalin and stored in 4°C overnight. The fixed lung tissues were transferred to 70% EtOH. The tissues were embedded with paraffin, and sliced sections were mounted onto slides for H&E staining. A minimum of five random fields were evaluated by microscopic projection and the NIH image program; alveolar size was estimated from the mean linear intercept (Lm) of the airspace as described previously (Wayne Rasband, NIH, Bethesda, MD).

### 4.5 Cell culture and reagents

hLMVEC were purchased from Lonza and were grown in EBM‐2 containing EGM‐2 supplement (Lonza; Basel, Switzerland). HUVEC were purchased from Yale Vascular Biology and Therapeutics core and cultured in M199 containing 20% FBS and Ec growth factor. For isolating primary MLEC, Ec (CD31+, CD45−) were isolated using Miltenyi Biotec magnetic columns or Tosyl‐activated Dynabeads and the autoMACS system using manufacturer‐supplied antibodies, as per the manufacturer's instructions. Briefly, minced lung tissues were digested with Collagenase Type A (1 mg/ml in DMEM/F12, Roche; Basel, Switzerland) with gentle agitation for 45 min at 37°C. The cell suspension was filtered through 70 μm cell strainers, and then centrifuged 300 × *g* for 3 min. Then, the supernatant was aspirated and discarded, and pellet were resuspended in 2 ml cold PBS with 0.1% BSA. The cell suspension was first depleted of magnetic CD45+ cells and then positively selected for magnetic CD31+ cells. Isolated MLEC were cultured in DMEM/F12 containing 20% FBS. All cells were plated to 80%–90% confluence and used between passages 3 and 5.

### 4.6 Construction of Lenti vectors and administration

Lenti‐silencing vectors with VE‐Cad promoter have been described previously (Zhang et al., 2013). p16^INK4a^‐knockdown vectors were designed by using target site 515–535 (GenBank accession NM_009877), 5′‐AGCGGAACGCAAATATCGCAC‐3′. We used Thermo Fisher Scientific Inc Scientific–Invitrogen's online RNAi Designer (Carlsbad, CA, USA) tool to design premiRNAstem loop/siRNA hybrid sequences per manufacturers’ instructions. Two oligos, 5′‐TGCTGAGCGGAACGCAAATATCGCACGTTTTGGCCACTGACTGACGTGCGATATGCGTTCCGCT‐3′ and 5′‐CCTGAGCGGAACGCATATCGCACGTCAGTCAGTGGCCAAAACGTGC GATATTTGCGTTCCGCTC‐3′, were annealed and ligated into *pcDNA6.2‐GW/EGFP‐miR* (Thermo Fisher Scientific Inc.) as per the manufacturer's instructions. The constructs were confirmed by sequencing. The *pcDNA6.2‐GW/EGFP‐miR*‐neg control plasmid was provided by Thermo Fisher Scientific Inc. The Lenti constructs of EGFP‐p16^INK4a^‐RNAi were constructed by first amplifying the fragments from *pcDNA6.2‐GW/EGFP‐miR* with primers: sense: 5′‐AGGCGCGCCTGGCTAACTAGAGAAC‐3′ and antisense: 5′‐GGAATTCTATCTCGAGTGCGGC‐3′, and by digestion of the PCR product with *AscI* and *EcoRI* enzymes. This fragment then was inserted into the *AscI* and *EcoRI* sites of FUW (Zhang et al., 2013), to generate lenti‐p16^INK4a^‐sil vectors. TLR4‐knockdown vectors were constructed with similar strategy and were designed using target site 550–570 (GenBank accession NM_021297.2), 5′‐GTACATGTGGATCTTTCTTAT‐3′ (Takyar et al., 2016).

### 4.7 Statistics

GraphPad Prism^®^ version 7.01 (GraphPad Software; La Jolla, CA) was used for data analysis. All experiments were performed in triplicate, and all data shown are means ± *SEM*. When only two groups were compared, statistical differences were assessed with unpaired 2‐tailed Student's *t* test. Otherwise statistical significance was determined using 2‐way ANOVA followed by Bonferroni multiple comparison test. *p* value less than 0.05 was considered statistically significant.

## CONFLICT OF INTEREST

None declared.

## AUTHOR CONTRIBUTIONS

C.H. and P.J.L. designed the research. S.‐J.K., C.H., P.S., Y.Z., T.A., and M.S. performed the experiments. P.S. prepared the human samples. P.S. and X.Z. generated and provided the TLR4−/−, Tie2‐TLR4‐Tg, and CC10‐TLR4‐Tg mouse line. Y.Z. generated and provided the lentivirus for Ub‐TLR4‐sil. J.‐N.M. generated and provided the lentivirus forEc‐p16^INK4a^‐sil and Ec‐TLR4‐sil. S.‐J.K., C.H., P.S., Y.Z., and P.J.L. assisted with data analysis. S.‐J.K., C.H., and P.J.L. prepared the figures and wrote the manuscript. S.‐J.K. performed the additional experiments and revised manuscript. P.Y., A.L., and T.P. performed micro‐CT analysis in lung tissues.
