# Supplementary material for: Endothelial toll‐like receptor 4 maintains lung integrity via epigenetic suppression of p16INK4a
Source: Aging Cell. 2019 Feb 20;18(3):e12914. doi: 10.1111/acel.12914 (PMC6516428; doi:10.1111/acel.12914)
Supplement: Supplementary file 1 [file ACEL-18-e12914-s001.docx]

**
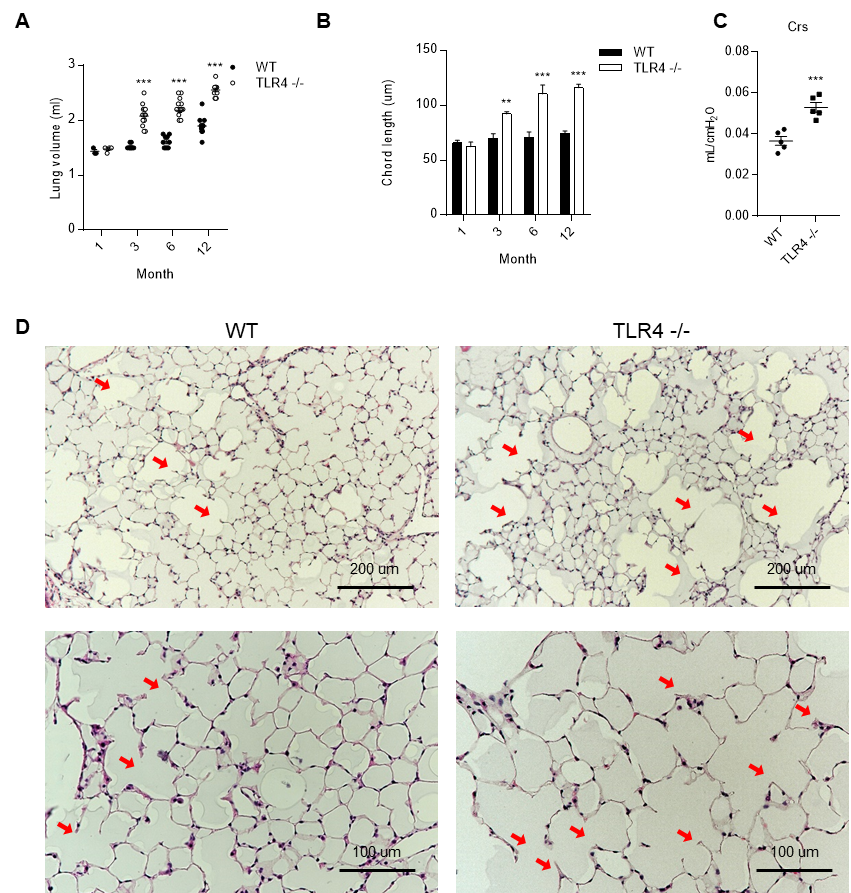
**

**Supplementary Figure 1. TLR4 deletion promotes age-related emphysema.**

(*A*) Lung volumes (ml) at 1, 3, 6 and 12 mo of age from WT and TLR4 -/- mice. (WT: n=3, *TLR4* -/-: n=4 in 1 mo, WT: n=10, *TLR4* -/-: n=18 in 3 mo, WT: n=10, TLR4 -/-: n=18 in 6 mo, WT: n=11, *TLR4* -/-: n=13 in 12 mo) ***p<0.001 vs age-matched WT. (*B*) Mean chord lengths at 1, 3, 6, and 12 mo of age from WT and TLR4 -/- mice. (n=3 per group) **p< 0.01, ***p<0.001 vs age-matched WT. (*C*) FlexiVent measurements of lung compliance (Crs) at 3 mo age in WT and TLR4 -/- mice. (n=5 per group). ***p<0.001 vs WT. (*D*) H&E stained lung sections taken from WT and TLR4 -/- mice at 3 mo age. Arrows point the enlarged airspace.

**
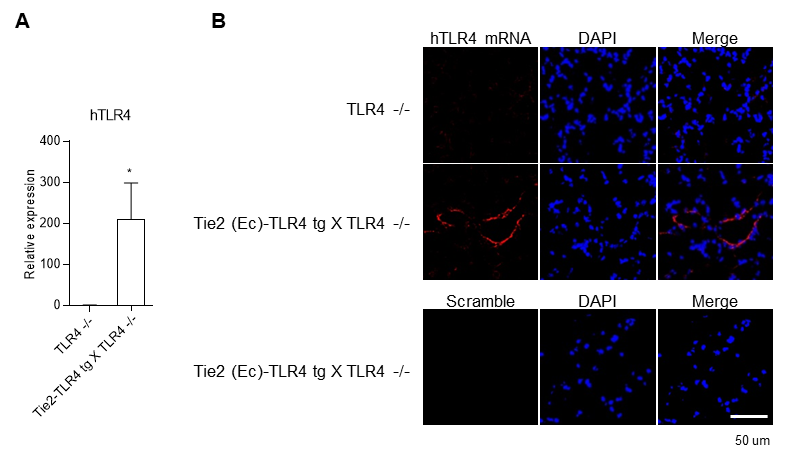
**

**Supplemental Figure 2. Restoration of endothelial (Ec) human TLR4 (hTLR4) in TLR4 -/- mice lungs.**

(A) Expression of *hTLR4* mRNA in lungs from TLR4-/- and Ec-TLR4 reconstit X TLR4-/- mice. (n=3 per group) *p< 0.05 vs TLR4 -/- (B) fluorescence *in situ* hybridization of hTLR4 in Ec-TLR4-reconstit X TLR4 -/- mice lung (Red: hTLR4, Blue: DAPI). Scale bar, 50 μm.

**Supplemental Figure 3. Epithelial (Epi) TLR4 partially prevents age-associated emphysema.**

Genetic, CC10-promoter driven restoration of lung Epi TLR4 (Epi-TLR4-reconstit) prevented lung enlargement inTLR4 -/- mice. Lung volumes (ml) at 3 mo of age from WT, Epi-TLR4-Tg, TLR4 -/-, and Epi-TLR4-reconstit X TLR4 -/- mice. (WT: n=4, Epi-TLR4-Tg: n=5, TLR4: n=5, Epi-TLR4-reconsit X TLR4 -/-: n=6) ***p<0.001.

**Supplemental Figure 4. Lung endothelial (Ec) TLR4 silencing in vivo.**

Lungs were harvested from WT mice at 2 mo after intranasal delivery of lentivirus that targeted all cells (Ub-TLR4-sil), Ec (Ec-TLR4-sil) or control (Ub-Con). Single cell suspensions were created by magnetic activated cell sorting (Miltenyi Biotec) with anti-CD45 and -CD31. *p< 0.05, **p<0.01 vs Ub-Con, n.s.: non-significant.


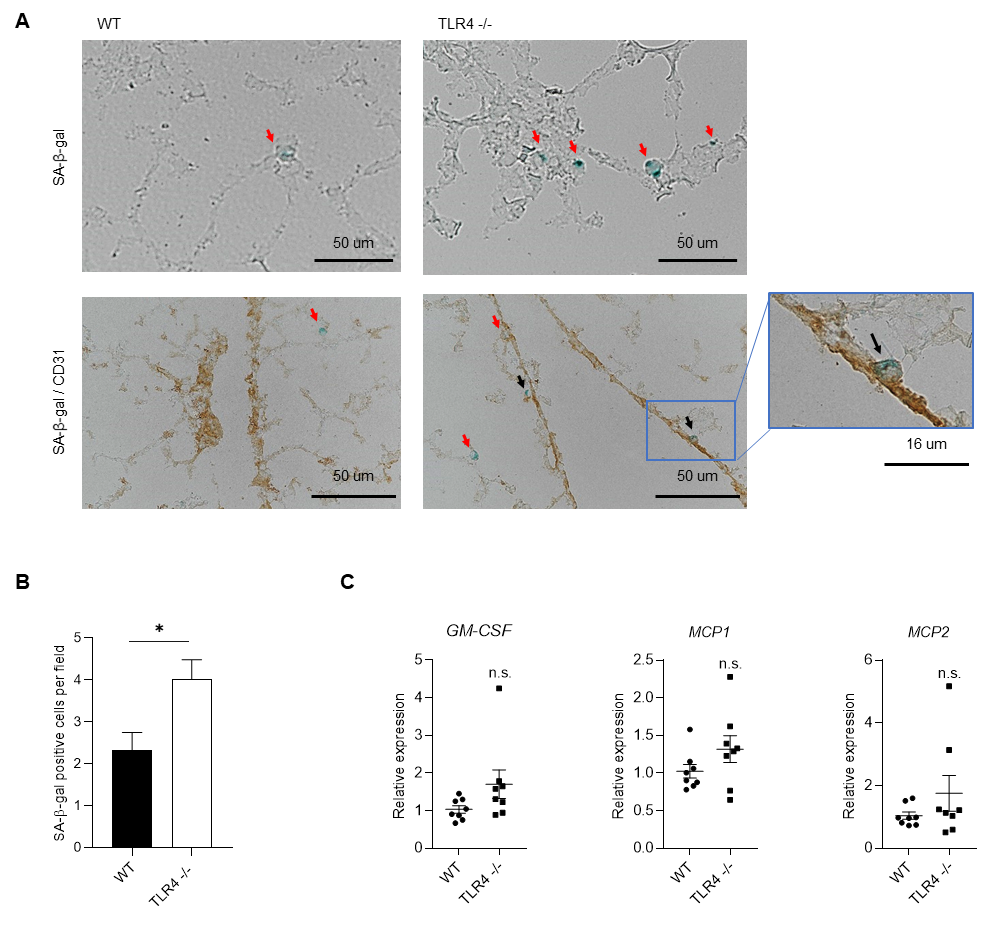


**Supplemental Figure 5. Senescence associated (SA)-β-gal activities and SA secretory phenotype (SASP) in TLR4 -/- mice.**

(*A*) SA-β-gal activities in lung tissues isolated from WT and TLR4-/- mice. The lung tissues were frozen and cut (thickness 6-8 µm). SA-β-gal activities (red arrows) and CD31 protein expression (brown) were measured by SA-β-gal analysis and immunohistochemistry, respectively. Co-localization of SA-β-gal and CD31 was indicated by black arrows. (*B*)

Quantification of the SA-β-gal positive cells. *p<0.05 vs WT. (*C*) Expression of *GM-CSF*, *MCP1* and *MCP2* mRNA in lung homogenate from WT and TLR4-/- mice. (n=8 per group). n.s. non-significant.

**
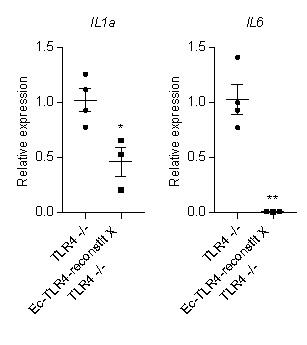
**

**Supplemental Figure 6. *IL-1α* and *IL-6* mRNA expressions in Ec-TLR4-reconstit X TLR4 -/- mice.**

Expression of *IL-1α* and *IL-6* mRNA in lung homogenate from TLR4 -/- and Ec-TLR4-reconstit X TLR4 -/- mice (WT: n=4, Ec-TLR4-reconsit X TLR4 -/-: n=3). *p<0.05, **p<0.01 vs TLR4 -/-.

**
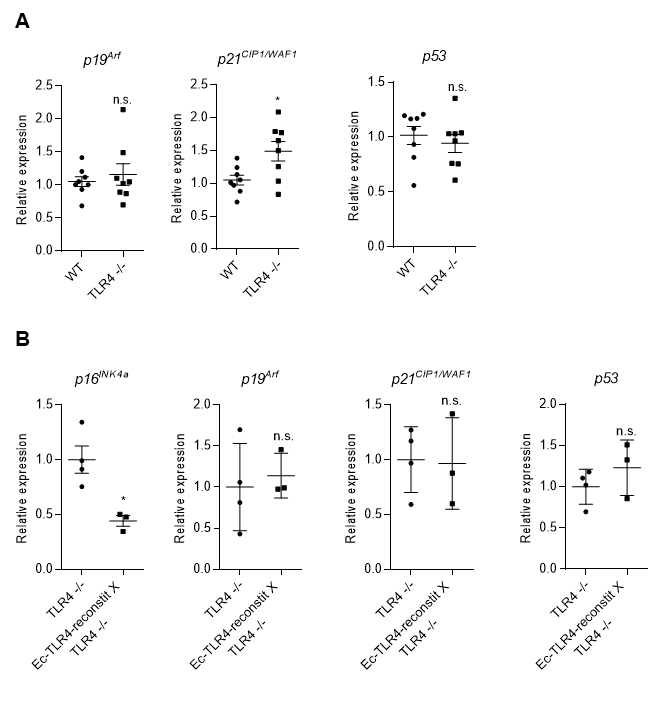
**

**Supplemental Figure 7. *p19^Arf^*, *p21^Cip1/Waf1^* and *p53* mRNA expressions in TLR4 -/- mice and Ec-TLR4-reconstit X TLR4 -/- mice.**

(*A*) Expression of *p19^Arf^*, *p21^Cip1/Waf1^* and *p53* mRNA in lung homogenate from WT and TLR4-/- mice. (n=8 per group). *p<0.05 vs WT, n.s.: non-significant. (*B*) Expression of *p16^INK4a^*, *p19^Arf^*, *p21^Cip1/Waf1^* and *p53* mRNA in lung homogenate from TLR4 -/- and Ec-TLR4-reconstit X TLR4 -/- mice (WT: n=4, Ec-TLR4-reconsit X TLR4 -/-: n=3). *p<0.05 vs TLR4 -/-, n.s. non-significant.

**
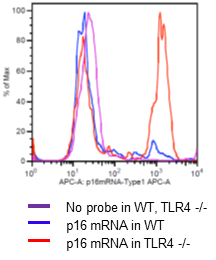
**

**Supplemental Figure 8. Single cell analysis for expression of *p16^INK4a^* mRNA in TLR4 -/- mice.**

Single cell analysis for expression of *p16^INK4a^* mRNA in WT and TLR4 -/- MLEC was performed using PrimeFlow™ RNA Assay. The cells were fixed, permeabilized, and intracellularly hybridized to label mRNA for *p16^INK4a^*.


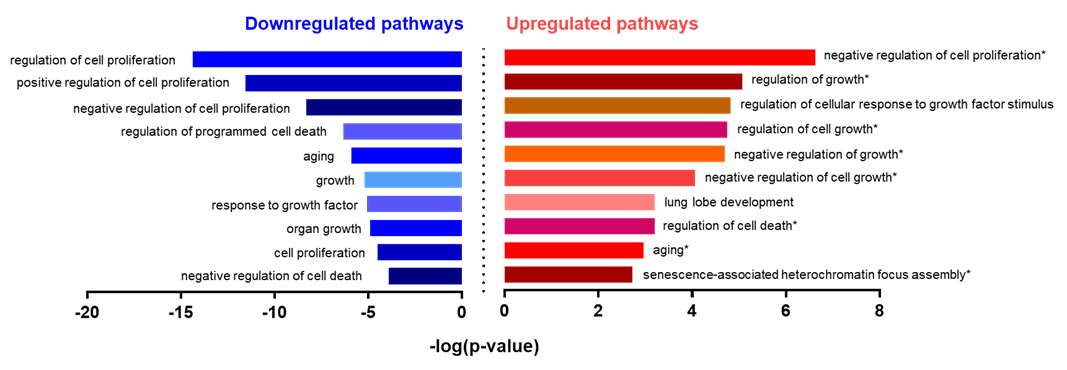


**Supplemental Figure 9. Micro array analysis in MLEC.**

Micro array analysis was conducted in Ec (CD45-CD31+) isolated from WT and TLR4-/- mice. The aging pathway related genes were upregulated in TLR4-/- MLEC such as *p16^INK4a^*, *HSP27*, *SOCS2*, *SCPX*, *HP*, *IGF-1*, *TGF-beta 3*, *FKHL1*, *G-protein alpha-o*, *AL3A1*, *LOXL2*, *TGF-beta receptor type I*, *angiotensinogen*, *VEGFR-1*, *SMPD*3, *collagen III*, *p14^ARF^*, and *ENO3*. In addition, positive regulators of cell proliferation were the most downregulated pathways in TLR4-/- MLEC, which is consistent with their hypo-proliferative phenotype. *p16^INK4a^* gene expression was upregulated in TLR4-/- MLEC, which confirms our findings. **p16^INK4a^* included pathways.

**
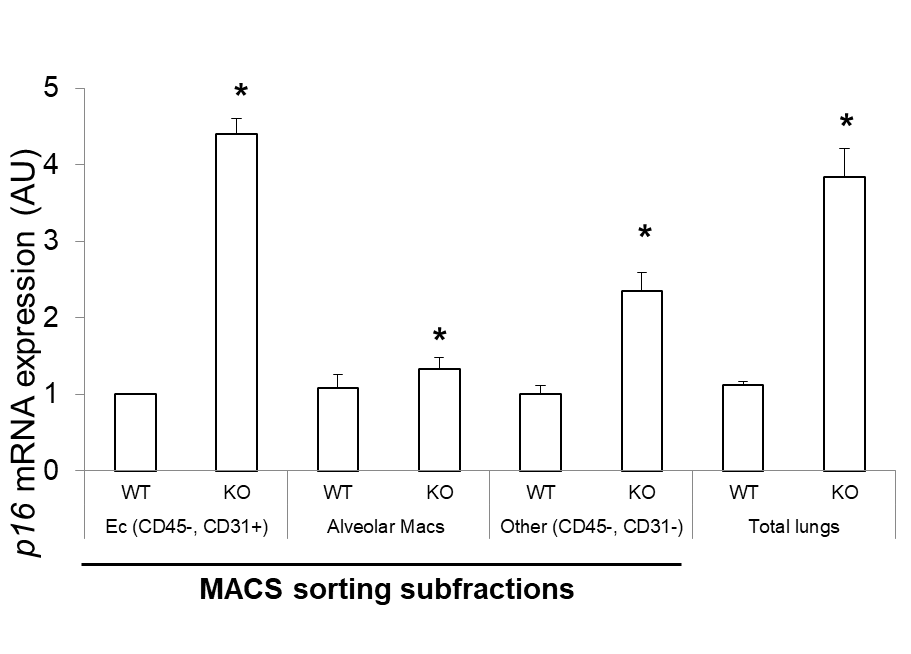
**

**Supplementary Figure 10. *p16^INK4a^* mRNA expression in lung subfractions.**

Lungs were harvested from WT and TLR4 -/- mice at 6 months old. Single cell suspensions were created by magnetic activated cell sorting (Miltenyi Biotec) with anti-CD45 and -CD31. Each subfraction was analyzed for *p16^INK4a^* mRNA with qPCR. *GAPDH* was detected as housekeeping control. (n=4 per group). *p<0.05 vs WT.

**
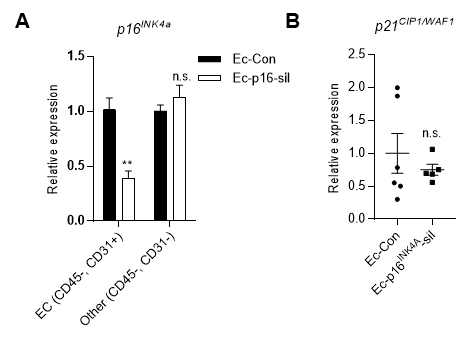
**

**Supplemental Figure 11. *p16^INK4a^* and *p21^Cip1/Waf1^* mRNA expression in endothelial cell (Ec)-p16^INK4a^-silencing mice (Ec-p16-sil) mice.**

Lungs were harvested from WT mice 3 mo after intranasal delivery of lentivirus that targeted Ec (Ec-TLR4-sil) or control (Ec-Con). (*A*) Single cell suspensions were created by magnetic activated cell sorting (MACs, Miltenyi Biotec) with anti-CD45 and -CD31.*p16^INK4a^* mRNA expression was measured by qPCR. **p<0.01 vs. Ec-Con. n.s.: non-significant. (*B*) Expression of *p21^CIP1/WAF1^* in lung homogenate from Ec-Con or Ec-p16-sil mice (Ec-Con X WT: n=6, Ec- p16^INK4a^-sil X TLR4 -/- n=5). **p<0.01 vs Ec-Con. n.s. non-significant.

**
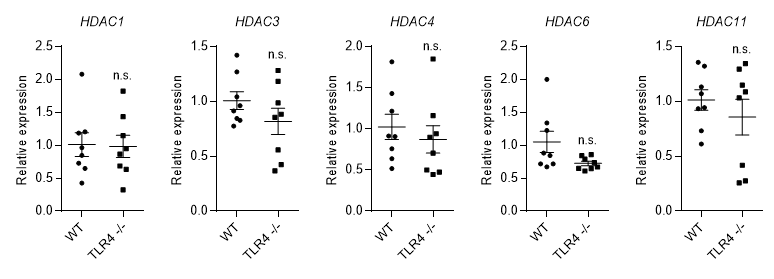
**

**Supplemental Figure 12. The mRNA expressions of *histone deacetylase (HDAC)1*, *3*, *4*, *6* and *11* were unchanged in TLR4 -/- mice.**

Expression of *HDAC1*, *3*, *4*, *6* and *11* mRNA in lung homogenate from WT and TLR4 -/- mice. (n=8 per group). n.s. non-significant.

**
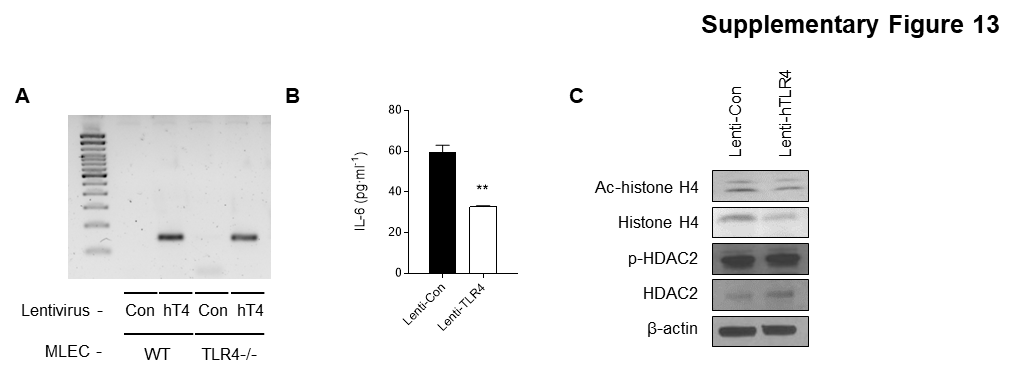
**

**Supplemental Figure 13. Restoration of human TLR4 (hTLR4) in TLR4 -/- MLEC.**

(*A*) *hTLR4* mRNA expression and (*B*) IL-6 production in TLR4 -/- MLEC with lenti-control or lenti-hTLR4. (*C*) Protein expressions of Ac-histone H4, histone H4, p-HDAC2 and HDAC2 in TLR4 -/- MLEC with lenti-hTLR4. **p<0.01 vs Lenti-Con.
