# Supplementary material for: Endothelial toll‐like receptor 4 maintains lung integrity via epigenetic suppression of p16INK4a
Source: Aging Cell. 2019 Feb 20;18(3):e12914. doi: 10.1111/acel.12914 (PMC6516428; doi:10.1111/acel.12914)
Supplement: Supplementary file 2 [file ACEL-18-e12914-s002.docx]

**Experimental procedures**

**Cell cycle analysis.** MLECs were grown to confluency and harvested with 0.25% Trypsin EDTA (25200-056, Gibco) and fixed with 70 % ethanol for overnight at -20°C. Then cells were resuspended in PI/RNase staining buffer (550825, BD Pharmingen^TM^) for 30 min at room temperature and analyzed by LSRII flow cytometry (BD Biosciences) and data was collected with DIVA software on LSR II and analyzed with FlowJo v10.1r7.

**Cell proliferation.** MLECs were plated onto 30 tissue culture 60 mm plates (triplicate samples for 16 days) at a density of 0.5 X 10^4^ cells per well. Cells were maintained in M199 with 20 % FBS and the media was replacedevery 48 h for 16 days. At the indicated time points, cells were resuspended and thoroughly mixed with an equal amount of 0.4% trypan blue solution (15250-061, Gibco), and subjected to cell counting by hemocytometer.

**Senescence associated (SA)-β-gal assay.** SA-β-gal staining (Cell signaling, 9860) was performed following manufacturer’s instructions. Briefly, 1 x10^5^ cells were plated in 6-well tissue culture plate and the next day cells were washed with PBS and fixed with 4% formaldehyde in PBS for 5 min at room temperature. Fixed cells were incubated with X-gal staining solution for 16 h at 37°C. Cells were washed with PBS, to stop staining, 50% glycerol added, and stored in 4°C until images were obtained. At least 20 different images were taken and more than 100 cells were counted per each cell plate. Lung tissues were frozen in Tissue-Tek O.C.T Compound (Sakura Finetek USA Inc) and cut into 6-8 μm sections. Staining and detection steps were performed as outlined for the cells.

**Immunohistochemistry.** The slides were incubated with 0.3% H_2_O_2_ solution and blocked with blocking solution, and then incubated with the indicated primary antibodies. The following day, the secondary antibodies were added to the slides and incubated for 1 h. After washing the slides with PBS, the slides were treated with DAB solution (Dako, Cat# K3468).

**siRNA and transfection.** Human siRNA against TLR4 and HDAC2 and non-targeting control (Invitrogen) were transfected with Lipofectamine RNAiMAX (13778-150, Life Technologies) per the manufacturer’s instructions. The cells were harvested 48 hours after the transfection.

**RNA extraction and RT-PCR.** The lung tissues or cells were homogenized in Qiazol (79306, Qiagen) and total RNA was extracted with the miRNeasy RNA isolation kit (Qiagen). Purified total RNA was reverse transcribed with the iScript cDNA Synthesis Kit (Bio-Rad). The mRNA expression levels of human and mouse transcripts were determined by quantitative real-time PCR with Sso-fast Evagreen supermix with low rox (Bio-Rad) on a 7500 Fast Real Time PCR (Thermo), per the manufacturer’s instructions. GAPDH was utilized for normalization. The following primers were used in RT-PCR: human *TLR4*, 5’-CCCTGAGGCATTTAGGCAGCTA-3’ and 5’-AGGTAGAGAGGTGGCTTAGGCT-3’, human *p16^INK4a^*, 5’-CTCGTGCTGATGCTACTGAGGA-3’ and 5’-GGTCGGCGCAGTTGGGCTCC-3’, human *HDAC2*, 5’-CTCATGCACCTGGTGTCCAGAT-3’ and 5’-GCTATCCGCTTGTCTGATGCTC-3’, and mouse *TLR4*, 5’-AGCTTCTCCAATTTTTCAGAACTTC-3’, and 5’-TGAGAGGTGGTGTAAGCCATGC-3’, mouse *p16^INK4a^*, 5’-CCCAACGCCCCGAACT-3’ and 5’-GCAGAAGAGCTGCTACGTGAA-3’, mouse *HDAC2*, 5’- GTTTTGTCAGCTCTCCACGGGT-3’ and 5’- CTTGGCATGATGTAGTCCTCCAG-3’, and mouse *IL-1α*, 5’-ACGGCTGAGTTTCAGTGAGACC-3’ and 5’-CACTCTGGTAGGTGTAAGGTGC-3’, and mouse *IL-6*, 5’-TACCACTTCACAAGTCGGAGGC-3’ and 5’-CTGCAAGTGCATCATCGTTGTTC-3’, and mouse *GM-CSF*, 5’-AACCTCCTGGATGACATGCCTG-3’, and 5’-AAATTGCCCCGTAGACCCTGCT-3’ mouse *MCP1*, 5’-GCTACAAGAGGATCACCAGCAG-3’, and 5’-GTCTGGACCCATTCCTTCTTGG-3’ mouse *MCP2*, 5’-GGGTGCTGAAAAGCTACGAGAG-3’ and 5’-GGATCTCCATGTACTCACTGACC-3’.

**PrimeFlow RNA Assay.** *p16^NI4a^* mRNA expression in single cell population of indicated MLEC were analyzed with PrimeFlow^®^ RNA Assay (Affymetrix, Ebioscience), per the manufacturer’s instructions. Cells were seeded at 1 × 10^6^ cells/well in 100 μl flow cytometry staining buffer per each well of V-bottom 96-well plate (Corning) and stained, on ice, with Fixable Viability Dye eFluor® 450 (65-0863-14, Affymetrix Ebioscience) and Alexa 488 Fluor-conjugated anti-mouse CD31 (553370, BD) for 30 min, then fixed and permeabilized with the staining set provided by the manufacturer. Samples were incubated with Alexa 647 Fluor-*p16^INK4a^* mRNA target probe for 2 h at 40 °C and samples were analyzed by LSRII flow cytometry (BD Biosciences). Data was collected with DIVA software on LSR II and analyzed with FlowJo.

**Western blot.** Cells were washed with ice-cold PBS in 3 times and lysed with 1X RIPA buffer (9806, Cell signaling) containing complete, mini EDTA-free (11836170001, ROCHE) and PhosSTOP (4906845001, Sigma Aldrich), and centrifuged at 15,000 rpm for 30 min at 4°C. The protein concentrations were measured with the micro BCA protein assay kit (23227, Thermo Scientific) and samples were boiled in a 95 °C heat block for 10 min and separated by SDS-PAGE. The separated proteins were electrically transferred to Trans-blot Turbo PVDF membrane (Bio-Rad) using the Trans-blot Turbo Transfer Starter System (1704155, Bio-Rad). The membranes were blocked with 5 % skim-milk in PBS-T (0.1 % Tween-20 in PBS) for 1 h at room temperature and incubated with the indicated antibodies. The membranes were washed with TBS-T and incubated with HRP-tagged secondary antibodies. The signal was detected using the ECL System (34096, Thermo Scientific). Each western blot is a representative of three independent experiments of triplicate samples. The antibodies used were: phospho-HDAC2 (Y461) (ab32117, Abcam), HDAC2 (5113, Cell Signaling), TLR4 (sc-293072, Santa Cruz), Acetyl-Histone H4 (K8) (ab15823, Abcam), Acetyl-Histone H4 (K5, K8, K12, K12) (PA1-84526, Thermo Scientific), Histone H4 (ab10158, Abcam), p16^INK4a^ (ab189034, Abcam), Actin (sc-47778, Santa Cruz).

**Immunohistochemistry and immunofluorescence.** Mice were anesthetized, and lungs were dissected from mice and immediately fixed in buffered formalin at 4°C overnight and processed for paraffin embedding and sectioned in Yale Pathology Tissue Services (YPTS) at 5 μm. Sections were de-paraffinized in Histo-Clear (National Diagnostics) and rehydrated via reverse ethanol series. Then, sections were incubated overnight at 4 °C with indicated antibodies including anti-TLR4 antibody (293072, Santa Cruz) and anti-CD31 (ab32457, Abcam) for human or anti-CD31 (553370, BD Bioscience) for mouse, and then detected with Alexa Fluor 568 donkey anti-goat IgG (A10037, Invitrogen), Alexa Fluor 568 goat anti-rabbit IgG (A11011, Invitrogen) and Alexa Fluor 488 goat anti-mouse IgG antibodies (A11001, Invitrogen) or Alexa Fluor 488 donkey anti-rat IgG antibodies (A21208, Invitrogen). DAPI (Sigma) was used to stain the nuclei. Immunofluorescence images were obtained on a Leica SP5 confocal at Yale Center for Cellular and Molecular Imaging (CCMI). Histological staining was performed on 5 μm sections by YPTS.

**mRNA *in situ* hybridization.** Paraffin-embedded tissue sections were de-paraffinized in Histo-Clear (National Diagnostics) and rehydrated via reverse ethanol series and digested for 10 minutes in 500 μg/mL Proteinase K (Sigma) followed by fixation in 4% PFA in PBS/0.1% Tween. The tissue was washed in PBS/0.1% Tween and pre-incubated in hybridization buffer (ENZ-33808, Enzo life Sciences) at 60 °C for 2 h and then incubated in hybridization buffer with DIG-labeled RNA probe for hTLR4 (DIG-5’-TGAAGATGATACCAGCACGACT-3’-DIG, custom LNA^TM^ mRNA detection probe designed at Exiqon). Probe signal amplified with TSA^TM^ Plus Cyanine 3 (NEL744001KT, Perkin Elmer) and DAPI (Sigma) was used to stain the nuclei. Images were obtained on a Leica SP5 confocal at Yale CCMI.

**Chromatin immunoprecipitation assay.** ChIP-IT^®^ Express Enzymatic Shearing Kits (Active motif) was used per the manufacturer’s protocol. Briefly, WT and TLR4 -/- MLEC were grown to 70 ~ 80 % confluency on 15 cm plates, and native protein-DNA complexes were cross linked by treatment with 1% formaldehyde for 10 min. Then, equal aliquots of isolated chromatin were subjected to immunoprecipitation with anti- Acetyl-Histone H4 (K8) (ab15823, Abcam) or anti-Acetyl-Histone H4 (K5, K8, K12, K12) (PA1-84526, Thermo Scientific) antibody or rabbit IgG control. PCR reactions of immunoprecipitated DNA were performed to validate Acetyl-Histone H4 (K8) or Acetyl-Histone H4 (K5, K8, K12, K12) binding on the p16^INK4a^ promoter, respectively. PCR primers used: #1: FWD: GATGGAGCCCGGACTACAGAAG and REV: GCTCCAAACAATGACAGAGAAC. PCR products were separated by gel electrophoresis and visualized by SYBRsafe (Invitrogen).

**Enzyme-linked immunosorbent assays (ELISA).** ELISA kits were used to measure IL-1α (R&D, MLA00) and IL-6 (R&D, M6000B). Assays were performed according to the manufacturer's instructions.

**Micro-computerized tomography (μCT) imaging of lung airspace**

Mice were sacrificed and the lungs were perfused with normal saline via the right ventricle. Lungs were excised from the body cavity, with heart and trachea attached, and fixed for 8 hrs with 10% neutral buffered-formalin perfused into the trachea at a pressure of 25 cmH_2_O. Lungs were then perfused and immersed into 5% phosphotungstic acid for 24 hours. Following agarose embedding, lung specimens were scanned on a Scano Medical µCT 50 high-resolution specimen scanner (UCSF Bone Imaging Core). X-ray energy was set at 45 kVp and 88 µA. Quantitative analyses were performed using the Scano µCT Evaluation Program. Regions of the lungs were delineated slice by slice to include the entire lung volume. Airspace volume was quantitated by the negative space within the lung specimen by applying an upper threshold for attenuation.
